# Sterile insect technique and *Wolbachia* symbiosis as potential tools for the control of the invasive species *Drosophila suzukii*

**DOI:** 10.1007/s10340-017-0944-y

**Published:** 2017-12-13

**Authors:** Katerina Nikolouli, Hervé Colinet, David Renault, Thomas Enriquez, Laurence Mouton, Patricia Gibert, Fabiana Sassu, Carlos Cáceres, Christian Stauffer, Rui Pereira, Kostas Bourtzis

**Affiliations:** 1 Department of Forest and Soil Sciences, Boku, University of Natural Resources and Life Sciences, Vienna, Austria; 2 UMR ECOBIO CNRS 6553, Université de Rennes, 1, 263 AVE du Général Leclerc, 35042 Rennes Cedex, France; 3 Institut Universitaire de France, 1 rue Descartes, 75231 Paris, Cedex 05, France; 4 Laboratoire de Biométrie et Biologie Evolutive, Univ. Lyon, Université Claude Bernard Lyon 1, CNRS, 69100 Villeurbanne, France; 5 Insect Pest Control Section, Joint FAO/IAEA Division of Nuclear Techniques in Food and Agriculture, Wagramerstrasse 5, PO Box 100, 1400 Vienna, Austria

**Keywords:** Biological control, Greenhouse, Incompatible insect technique, Integrated pest management, Spotted wing *Drosophila*

## Abstract

*Drosophila suzukii*, a vinegar fly originated from Southeast Asia, has recently invaded western countries, and it has been recognized as an important threat of a wide variety of several commercial soft fruits. This review summarizes the current information about the biology and dispersal of *D. suzukii* and discusses the current status and prospects of control methods for the management of this pest. We highlight current knowledge and ongoing research on innovative environmental-friendly control methods with emphasis on the sterile insect technique (SIT) and the incompatible insect technique (IIT). SIT has been successfully used for the containment, suppression or even eradication of populations of insect pests. IIT has been proposed as a stand-alone tool or in conjunction with SIT for insect pest control. The principles of SIT and IIT are reviewed, and the potential value of each approach in the management of *D. suzukii* is analyzed. We thoroughly address the challenges of SIT and IIT, and we propose the use of SIT as a component of an area-wide integrated pest management approach to suppress *D. suzukii* populations. As a contingency plan, we suggest a promising alternative avenue through the combination of these two techniques, SIT/IIT, which has been developed and is currently being tested in open-field trials against *Aedes* mosquito populations. All the potential limiting factors that may render these methods ineffective, as well as the requirements that need to be fulfilled before their application, are discussed.

## Key message

- *Drosophila suzukii* has invaded the Americas and Europe, and it has become a significant global pest of a wide variety of soft fruit crops.
- We review current knowledge on management practices used so far to control *D. suzukii* and discuss innovative biological control methods.
- The SIT can be used as part of an area-wide integrated pest management (AW-IPM) strategy to control *D. suzukii* in greenhouses and other confined locations.
- A combined SIT/IIT strategy should be considered as a contingency plan.

## Introduction

**Fig. 1:**
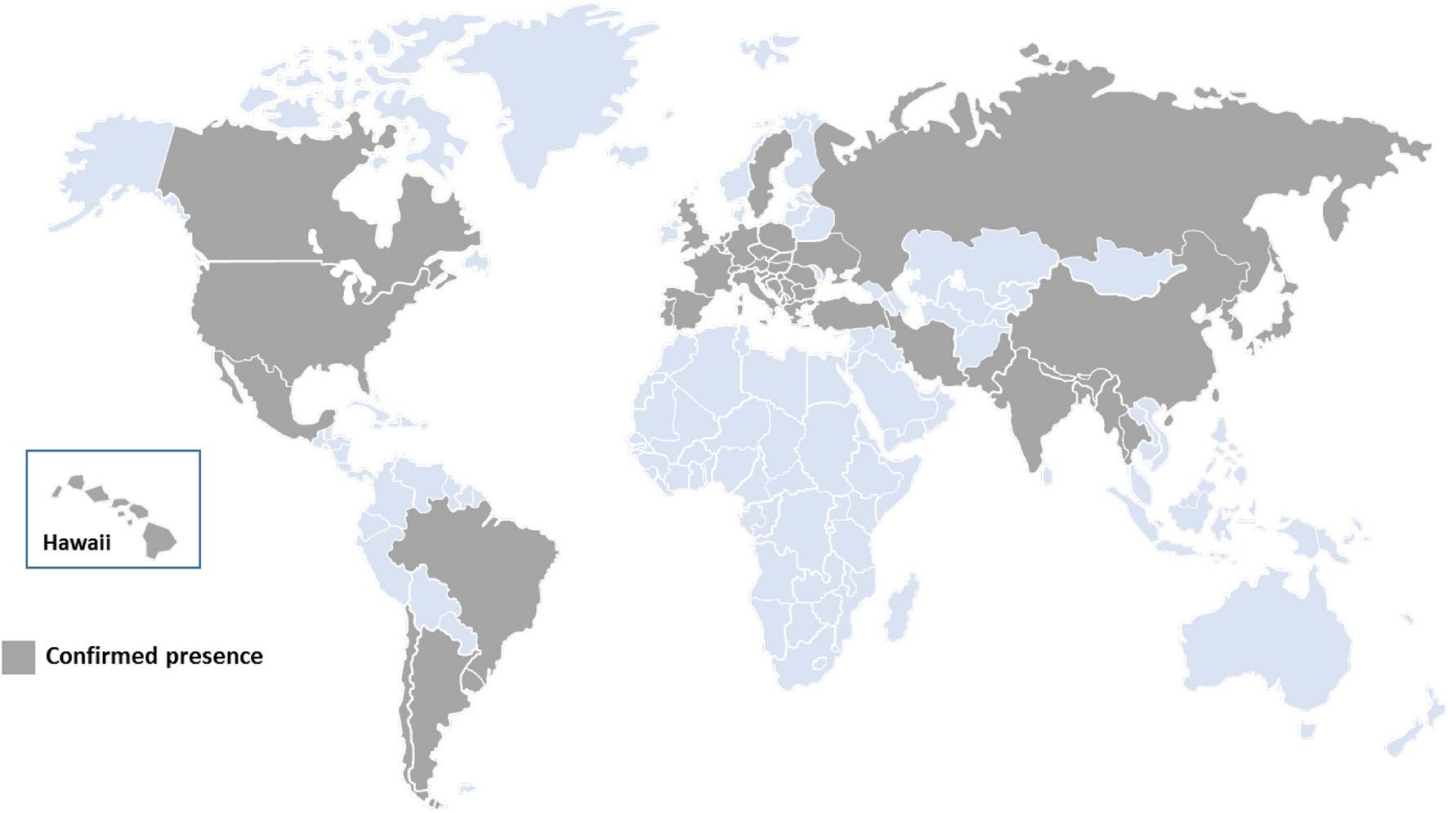
Worldwide confirmed distribution of *D. suzukii* (as of August 2017)

Invasive species have been recognized as important threats of biodiversity and cause substantial yield and revenue losses in agricultural systems (Bolda et al. 2010; Goodhue et al. 2011; Pimentel et al. 2000, 2005). The spotted wing *Drosophila* (SWD), *Drosophila suzukii,* was originally described by Matsumura in Japan in 1931. Recently, *D. suzukii* has invaded North and South America (Bolda et al. 2010; Deprá et al. 2014) and Europe (Calabria et al. 2012; Cini et al. 2012). The most probable source for the western North American populations seems to be southeast China and Hawaii, while European populations are probably originated from northeast China, with evidence of limited gene flow from eastern USA as well (Fraimout et al. 2017). Members of the *Drosophila* genus are not generally considered as pests since their larvae are primarily developed on damaged or rotting fruits. Nevertheless, *D. suzukii* infests healthy ripening fruits while still on the plant. *Drosophila suzukii* larvae consume the fruit pulp inside the fruits rendering them unmarketable and decreasing the processed fruit quality (Walsh et al. 2011). Moreover, the wounds created on the infested fruits during oviposition provide access to secondary fungal or bacterial infections leading to additional fruit tissue collapse (Asplen et al. 2015; Cini et al. 2012; Goodhue et al. 2011; Ioriatti et al. 2015; EPPO 2010; Walsh et al. 2011). *Drosophila suzukii* has a wide range of hosts including both cultivated fruits and wild plants (Asplen et al. 2015; Diepenbrock and Burrack 2017; Grant and Sial 2016; Poyet et al. 2014, 2015). This species has thus become a significant worldwide pest (Fig. 1) of a large variety of commercial fruit crops. *Drosophila suzukii* is differentiated from other drosophilids based on two key morphological traits: (a) females have an enlarged serrated ovipositor which enables them to infest and cause physical damage to the ripening fruit and (b) males are characterized by a dark spot on the leading edge of the wings.

Significant damage has been observed in several commercial soft fruits, such as blackberries, blueberries, cherries, raspberries, strawberries, tomatoes, grapes, cherries, figs, kiwis (Ioriatti et al. 2015; Lee et al. 2015; Poyet et al. 2015; EPPO 2010; Rota-Stabelli et al. 2013; Tochen et al. 2014). Recent studies have shown that specific host fruits favor the oviposition and development of larvae, while temperature plays a crucial role on *D. suzukii* development, survival and fecundity (Ioriatti et al. 2015; Lee et al. 2011a, b; Tochen et al. 2014).

First records of *D. suzukii* in North America date back to 2008 (Hauser and Gaimari 2009; Walsh et al. 2011). In Europe, this fly was first recorded in Spain in autumn 2008 (Calabria et al. 2012) and in North Italy in 2009 (Grassi et al. 2011). By the end of 2010, *D. suzukii* had colonized the Western and Eastern USA, Canada, and most of the Mediterranean regions (Rota-Stabelli et al. 2013). Latest records report the presence of this pest in additional countries such as Austria, UK, Belgium, Germany, Hungary, Romania Turkey, Ukraine, Brazil, Chile, Argentina and Uruguay (Asplen et al. 2015; Calabria et al. 2012; Cini et al. 2012; Chireceanu et al. 2015; Deprá et al. 2014; Lavrinienko et al. 2017; Lengyel et al. 2015; Lue et al. 2017; Orhan et al. 2016; Servicio Agrícola y Ganadero (SAG) 2017; Vilela and Mori 2014). The expansion of *D. suzukii* in North and South America and in Europe has been very fast and widespread (Adrion et al. 2014; Lasa and Tadeo 2015). Adults of *D. suzukii* demonstrate a high dispersal potential, which is mainly attributed to the increasing global trade and the pest’s invasion behavior (Calabria et al. 2012; Lengyel et al. 2015; Rota-Stabelli et al. 2013). From an ecological standpoint, *D. suzukii* adapts easily to environments with high humidity and moderate temperatures (EPPO 2010; Ometto et al. 2013). These environments allow the pest to overwinter when fruit resources are not available and low temperatures are not optimal for fermentation and fly activity (Ometto et al. 2013; Rota-Stabelli et al. 2013). Absence of natural predators and/or effective parasitoids against *D. suzukii*, as well as competitors for fresh fruits (Chabert et al. 2012; Rota-Stabelli et al. 2013), facilitates its establishment in the invaded habitats. Finally, *D. suzukii* shows a high reproductive rate and rapid developmental rate which results in 7–15 generations per year, depending on the weather conditions (Tochen et al. 2014).

*Drosophila suzukii* has caused substantial yield and revenue losses in agricultural systems. In the USA (California, Oregon and Washington), losses were estimated around $511.3 million annually at 20% damage of strawberries, blueberries, raspberries, blackberries and cherries in 2008 (Bolda et al. 2010; Goodhue et al. 2011; Walsh et al. 2011). Increased costs owing to monitoring and management programs of the pest could also decrease revenue. Regulatory restrictions applied on shipments from infested areas (e.g., quarantine) could lead to significant economic impact. Residual pesticide levels exceeding tolerated limits in fruits from infested areas or postharvest treatments may also lead to rejection of exported fruits, thus limiting fruit market exploitation (Goodhue et al. 2011; Walsh et al. 2011). As a result, global economic loss for fruit production areas is potentially huge.

Considering the significant and rapidly growing agricultural costs generated by the worldwide invasion of *D. suzukii*, we review the knowledge gained so far about the tools that have been deployed to combat this pest. Following a brief review of the current management practices, the classical biological control procedures, we discuss innovative biological control methods and their potential as management solutions for facing the challenge posed by *D. suzukii*.

## Pest management: current state and perspectives

*Drosophila suzukii* has become a key economic pest, and therefore the development of efficient monitoring and management tools is deemed indispensable. Understanding the pest’s invasion mechanisms and gaining higher resolution on its biology are needed to improve management practices (Bahder et al. 2015; Lee et al. 2011a, b). The diverse array of alternate host fruits used by *D. suzukii* and its extreme polyphagy behavior contribute to its persistence in distinct geographic areas, thus escalating its effective management into a challenge (Diepenbrock and Burrack 2017; Lee et al. 2011a, b). Currently several control methods, such as the classical chemical control, are applied worldwide to manage this pest. Nevertheless, these methods proved to be either non-effective or non-cost-effective or with limited applicability due to regulatory restrictions. In the following sections, we address biological and innovative pest management approaches and discuss their application potential and drawbacks.

## Biological control

Given legitimate concerns over the risks and limitations of using a chemical control method, research efforts have already been focused on the development of environmentally sound and sustainable methods. There is a wide variety of biocontrol agents including fungi, bacteria, viruses and natural enemies of the pest that could be employed in the control programs for *D. suzukii*.

### Natural enemies

Natural enemies of insect pests are endemic species that occur abundantly in agricultural fields. Natural enemies including pathogens, predators and parasitoids can be specialists or generalists, and they can induce a high level of mortality in their hosts (Flint and Dreistadt 1998). Biological control approaches based on arthropod natural enemies are currently studied and developed worldwide.

Parasitoid species are insects attacking other arthropods in the egg, larval or pupal developmental stages. They develop inside, or on the surface of the egg, larvae or pupae, and consume the host tissues during their development (Godfray 1994). Various *Drosophila* species are subjected to strong selective pressures by egg, larval and pupal parasitoids which play a key role in their population suppression. Most studies agree that *Drosophila* parasitoids induce a high rate of mortality on their host populations although the level of parasitism varies with breeding sites, local conditions and seasons (Fleury et al. 2009). Studies on natural parasitoid enemies of *D. suzukii* in its invaded regions have shown that parasitism rates are limited, and thus their use is non-efficient for population suppression (Chabert et al. 2012; Daane et al. 2016; Miller et al. 2015). This is attributed to the fact that *D. suzukii* exhibits a high level of resistance to the majority of the larval parasitoids tested, associated to a highly efficient cellular immune system (Poyet et al. 2013; Kacsoh and Schlenke 2012). However, a recent study by Rossi Stacconi et al. (2015) showed that Italian populations of *Leptopilina heterotoma* (Hymenoptera: Figitidae) were able to overcome the encapsulation process by *D. suzukii* under standard laboratory conditions, a fact that could be attributed to the high virulence level of the wild parasitoid population collected in Italy (Rossi Stacconi et al. 2015). In contrast, generalist pupal parasitoids (e.g., *Pachycrepoideus vindemiae* and *Trichopria c.f. drosophilae*) were able to develop on *D. suzukii*, at least under laboratory conditions (Chabert et al. 2012; Daane et al. 2016; Miller et al. 2015; Rossi Stacconi et al. 2015; Wang et al. 2016). Kacsoh and Schlenke (2012) used a diverse panel of parasitoid wasps and found that *D. suzukii* was able to survive infection due to the production of a constitutively high hemocyte level. High hemocyte loads are involved in encapsulation of parasitoid eggs and enable *D. suzukii* larvae to produce a vigorous immune response (Kacsoh and Schlenke 2012). Moreover, spontaneous parasitization of *D. suzukii* by *P. vindemiae* has been recently reported suggesting a gradual adaptation of the local fauna to the new invader (Rossi-Stacconi et al. 2013). In field-sampling studies in Japan, three larval endoparasitoids were reported to develop on *D. suzukii*, *Asobara japonica* (Hymenoptera: Braconidae), *G. xanthopoda* and *Leptopilina japonica japonica* (Hymenoptera: Figitidae) (Daane et al. 2016; Kasuya et al. 2013; Mitsui et al. 2007). Ongoing studies on biological control of *D. suzukii* by parasitoids are now focusing on the description of the efficiency of parasitoid species in the native area.

Progressively, government regulations require the development of host-specialized biological control agents. Consequently, a diverse array of other natural enemies including predators, entomopathogenic fungi and nematodes which are commercially available was evaluated for their ability to reduce *D. suzukii* adults and larvae survival (Woltz et al. 2015; De Ro 2016). Our current knowledge suggests that *D. suzukii* suppression by these enemies was insufficient due to low predation and infection rates, low residual activity or decreased efficiency in field trials. Taken together, extensive field studies and detailed evaluations are required to identify a novel strategy based on introduction and establishment of natural enemies of *D. suzukii* from its native range for a long-term control and determine their effectiveness and safety with regard to nontarget species.

## Innovative biological control methods

### Sterile insect technique (SIT)

**Fig. 2:**
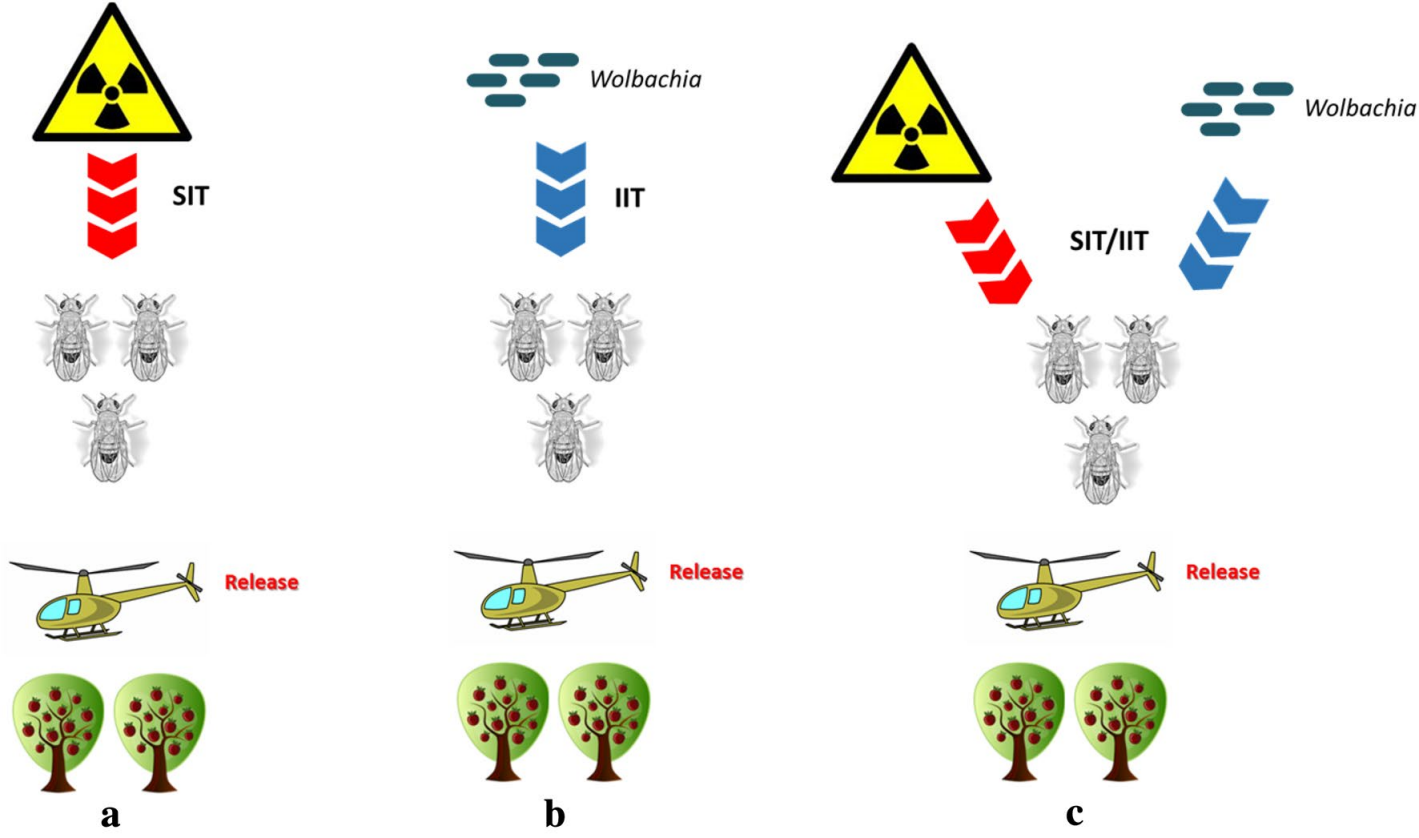
**a** Sterile insect technique (SIT). Males are sterilized by the application of irradiation, **b** the incompatible insect technique (IIT). Males are sterilized by *Wolbachia* trans(infection), **c** combination of SIT and IIT. Male sterility is due to both irradiation and *Wolbachia* infection. In all three cases (**a**–**c**), males are released in the field to sterilize the wild females of the targeted population

The sterile insect technique (SIT) is a species-specific and environment-friendly method of pest population suppression or eradication (Fig. 2a). The SIT relies on repetitive releases of mass-produced sterile insects (Dyck et al. 2005). The method is based on the sterilization of males (although releases of both sterile males and females have been successfully used), mainly using ionizing radiation which causes dominant lethal mutations in the sperm. In brief, the SIT comprises the following steps: (a) the target species is mass reared, (b) males are separated, when feasible, and sterilized and (c) released in the target area. A sufficient number of sterile males to create an overflow ratio over a period of time are released, and they are expected to compete with wild males and mate with wild females (Dyck et al. 2005). Mating results in infertile eggs and the developing zygotes die during early embryogenesis, thus inducing sterility in the wild females. Therefore, over time, the target population declines or it is potentially eradicated (Knipling 1979).

Effectiveness of SIT is undoubtedly associated with the ability of irradiated males to compete with wild males for mating with wild females. The competitiveness of released sterile males might be impacted by the insect strain and the rearing method, radiation sterilization, marking, stress during cold storage, shipment to the release site and release procedure (Dyck et al. 2005). Therefore, it is essential that the impact of the domestication, irradiation dose, as well as all other components of the SIT package, on emergence rate, adult longevity and mating competitiveness is checked and assessed prior to field application (Dyck et al. 2005; Zhang et al. 2016). Performance of sterile males is not the only critical factor that could affect success of SIT. In any SIT program, the number of released sterile males must surpass the number of wild males in the release area to compensate any negative effect associated with domestication, mass rearing, storage and their overall handling so that they mate with wild females allowing introduction of sufficient sterility into the wild population (Barnes et al. 2015; Dyck et al. 2005; Vreysen 2006). The same is true for any population suppression program, no matter if it is based on classical genetic, transgenic or symbiont-based approaches.

Apart from being an environmentally sound biological control approach, the SIT can be easily integrated with other biological control strategies (parasitoids, predators and pathogens). It is a species-specific method, and the release can be performed from the air thus overcoming any topography limitations. Successful development and application of an SIT operational program depends on: (a) the target population being at low levels; (b) extensive knowledge on the genetics, biology and ecology of the target pest being available before the application; (c) mass-rearing facilities being available and capable of providing large numbers of high-quality sterile insects; (d) a release technology having been developed, and the sterile individuals being efficiently monitored; (e) the releases being applied on an area-wide basis covering the whole pest population and (f) the released sterile individuals not causing any side effects on humans or the environment (Barnes et al. 2015; Vreysen 2006).

The SIT has proven to be a powerful control tool when applied as part of an area-wide integrated pest management (AW-IPM) approach for the creation of pest-free areas or areas of low pest prevalence (Vreysen 2006). The use of the SIT was initially put into practice in the USA, and it was subsequently developed and applied worldwide by the Joint Food and Agriculture Organization of the United Nations/International Atomic Energy Agency (FAO/IAEA) Programme on Nuclear Techniques in Food and Agriculture and collaborators (Barnes et al. 2015; Bourtzis and Robinson 2006; Dyck et al. 2005; Vreysen 2006).

The SIT has been refined over many decades, and renewed interest has recently emerged to use it for the population control of human diseases vectors (Bourtzis et al. 2016). Several successful applications of the method have been reported worldwide including the control of key insect pests such as the screwworm fly, the tsetse flies, fruit flies, Lepidoptera (moths) and disease vectors of livestock and humans (Barnes et al. 2015; Bourtzis et al. 2016; Calla et al. 2014; Lees et al. 2015; Munhenga et al. 2016; Pereira et al. 2013; Vreysen et al. 2014; Zhang et al. 2015a). The majority of the SIT programs have been applied for the control of fruit fly species as they represent one of the major insect groups of economic importance (FAO/IAEA 2013, https://nucleus.iaea.org/sites/naipc/dirsit/; Pereira et al. 2013). The acknowledged deliverables of these applications have encouraged researchers to focus on ways to improve the performance of mass-reared sterile males as well as the handling and release methods. Significant knowledge acquired from SIT applications on the genera *Anastrepha*, *Bactrocera* and *Ceratitis* can be transferred partly or entirely to other insect pest species control programs (Pereira et al. 2013).

The rapid dispersal of *D. suzukii* and its subsequent impacts on crops encourage the development of a biocontrol method with a SIT component. Radiation biology experiments are currently ongoing on *D. suzukii,* and first results have shown that X-ray radiation can inhibit the development of all stages (egg, larva, pupa and adult) of *D. suzukii* and induce adult sterility (Follett et al. 2014; Kim et al. 2016). Nevertheless, there are some reasonable concerns about the feasibility of SIT for this pest considering its high fecundity and the recurrent immigration of flies into the crop that are not completely confined. The short generation time of *D. suzukii* indicates that SIT management should be intensive, otherwise there is a risk that the population will recover rapidly. In addition, control of large field populations of *D. suzukii* poses an extra challenge for SIT. In our opinion, greenhouses and other confined locations seem to provide an ideal environment for the biocontrol of *D. suzukii* by using the SIT. The exclusion netting high tunnels could be promising candidates for the implementation of SIT. Recent studies on plastic- and mesh-covered tunnels have shown that *D. suzukii* populations are significantly decreased in these confined areas, not only due to their physical exclusion, but also because of the unfavorable microclimate that is created in these locations (Rogers et al. 2016). Although complete exclusion is not achievable solely by this technique, its combination with SIT could increase the biocontrol levels of *D. suzukii*, thus limiting the use of insecticides. An additional challenge is that an adequate sexing system is not available for *D. suzukii,* and this means that both males and females will be included in the mass-reared and released flies. Bisexual SIT has been successfully used in the past; however, male only releases have been shown to be by far more cost-effective and efficient (Rendon et al. 2000).

### Incompatible insect technique (IIT)

*Wolbachia* is a widespread endosymbiont of arthropods and filarial nematodes. *Wolbachia* can act as both a parasite and a mutualist, but it is best known for its ability to manipulate their host reproduction (for a review see Werren 1997). Four distinct reproductive alterations have been described in arthropods: feminization, parthenogenesis, male killing and cytoplasmic incompatibility (Saridaki and Bourtzis 2010; Werren et al. 2008). Collectively, these phenotypes are commonly referred to as “reproductive parasitism,” and they increase the frequency of infected females in the host population either by inducing a female-biased sex-ratio in the offspring of infected females, or by reducing the female production by uninfected females (Engelstädter and Hurst 2009).

Among the reproductive abnormalities associated with *Wolbachia* infections, cytoplasmic incompatibility (CI) is the most prominent one. *Wolbachia* induces modification of the paternal nuclear material which results in failure of progeny to develop unless the same *Wolbachia* strain(s) is/are present in the egg and exert(s) the respective rescue function(s) (Bourtzis et al. 1998; Werren 1997; Zabalou et al. 2008). *Wolbachia*’s ability to manipulate the host reproductive system along with its great pandemic has largely been recognized as potential environmental-friendly biocontrol agent. The incompatible insect technique (IIT) employs the symbiont-associated (e.g., *Wolbachia*) reproductive incompatibility as a biopesticide for the control of insect pests and disease vectors (Fig. 2b). The approach is quite similar to SIT and includes repeated, inundative releases of sterile males in the targeted field population (Berasategui et al. 2016; Bourtzis 2008; Zabalou et al. 2004, 2009). Intensive research in IIT has been performed for several insect pests and disease vectors including *Ceratitits capitata*, *Rhagoletis cerasi*, the tsetse fly, *Culex pipiens*, *Aedes albopictus* and *Culex quinquefasciatus* (Alam et al. 2011; Atyame et al. 2011, 2015; Bourtzis et al. 2014; Neuenschwander et al. 1983; Zabalou et al. 2004, 2009; Zhang et al. 2015a), and significant attempts to use IIT against wild populations of disease vectors have been applied for the mosquito *Aedes polynesiensis* (O’Connor et al. 2012).

One of the main points of this technique is that, contrary to SIT that allows both sexes to be released as long as they are sterile, this is not possible for IIT which requires strict male release. Indeed, the accidental release of females infected by *Wolbachia* may result in the replacement of the targeted population by a population carrying the *Wolbachia* infection. Providing that IIT produced females are compatible with the wild males, the success of IIT could be compromised, since the *Wolbachia*-infected females would be compatible with either the wild or the released males (Berasategui et al. 2016; Bourtzis 2008; O’Connor et al. 2012). Therefore, IIT requires the development of an efficient method for sexing in order to strictly release infected males. Sexing can be achieved by different techniques like phenotypic sorting or genetic-sexing methods based on classical genetic or molecular methods. However, these separation methods are not available for all target species. In addition, there are concerns about the use of GMOs in the European Union.

Although *Wolbachia* infections are highly prevalent in the drosophilids, CI is not induced by all *Wolbachia* strains (Hoffmann et al. 1996). However, there is evidence that *Wolbachia* can engage in mutualistic relationships (Zug and Hammerstein 2015) and it has been shown to provide a broad spectrum of beneficial effects to its native hosts including protection against viral, microbial, fungal pathogens and parasitoids (Bian et al. 2013; Cattel et al. 2016b; Fytrou et al. 2006; Kambris et al. 2010; Martinez et al. 2012, 2014; Moreira et al. 2009; Teixeira et al. 2008; Zindel et al. 2011; Zug and Hammerstein 2015) and increase in host longevity and fecundity (Zug and Hammerstein 2015) which probably explains its pandemic nature (LePage and Bordenstein 2013).

Previous studies reported that *D. suzukii* is infected with a strain of *Wolbachia* called *w*Suz that is present in intermediate prevalence in European and American populations (Cattel et al. 2016a; Hamm et al. 2014; Ometto et al. 2013; Siozios et al. 2013). *w*Suz does not induce a significant level of CI in *D. suzukii* (Cattel et al. 2016a; Hamm et al. 2014). However, *Wolbachia* endosymbionts inducing CI can be introduced into a novel host, either by back-crossing experiments or by transinfection, and express high levels of CI (Zabalou et al. 2008). This concept has been studied in insect pests and disease vectors for the suppression of natural populations (Laven 1967; O’Connor et al. 2012; Zabalou et al. 2004; Zhang et al. 2015b). Research work has been performed in this field for *D. suzukii,* and two *Wolbachia* strains have been identified as potential candidates for developing the IIT in *D. suzukii*. Both strains were identified using the transinfection approach, and they induce a very high level of CI in this pest regardless of the presence of *w*Suz in females (Mouton et al., personal communication). However, it is critical to address any questions related to host fitness and mating competitiveness of the *Wolbachia*-infected *D. suzukii* males under semi-field conditions prior to the deployment of this approach to a large-scale operational program (O’Connor et al. 2012; Zhang et al. 2015a). As discussed above for SIT, male competitiveness in an IIT program may be impacted by the rearing methods and processes, cold storage, shipping and release approaches, but also by the introduction of new *Wolbachia* strains as in the case of transinfected lines. A potential IIT application would first and foremost require a thorough biological characterization of the host–bacterial symbiotic association. The *Wolbachia* strain and the host nuclear background are important factors for the expression of CI. Previous studies have suggested that the genetic background of the host is actively involved in the expression of different *Wolbachia* phenotypes, affecting also the *Wolbachia* density. This means that ideally the genomic background of the mass-reared insects should be the same with the one in the target field population. In addition, infection with one or more *Wolbachia* strains could impact the fitness and sexual behavior of host insects, leading in negative effects on the host sexual competitiveness and fitness traits (Bourtzis et al. 2014; Mouton et al. 2007). Given the above, mass-reared insect lines should be evaluated for any potential impacts of the *Wolbachia* symbiont and the genetic variability of the host before including IIT in an integrated control approach.

### Combination of SIT/IIT for *D. suzukii* management

A promising alternative approach for the biological control of *D. suzukii* is coupling SIT with IIT (Fig. 2c). In general, female insects are more sensitive to radiation than male insects in terms of the induction of sterility, and it may be possible to identify a minimum dose of radiation that leads to complete sterility in females (Bourtzis and Robinson 2006; Zhang et al. 2015a, 2016). As a result, any accidentally released *Wolbachia*-infected females will be sterile and the risk of population replacement is reduced (Bourtzis et al. 2014, 2016; Brelsfoard et al. 2009; Lees et al. 2015). In such a system, the released cytoplasmically incompatible males could also receive a low dose of radiation to ensure complete sterility of females that were not removed. In this case, the sterility of released males would be due to both *Wolbachia* and irradiation, while the female sterility would only be caused by irradiation. Stress accumulated throughout the rearing, handling, storage, transport and release processes may affect the biological quality of the released males (Dyck et al. 2005). Less competitive males in the field would result in lower induction of sterility in the field females. The combination of SIT with IIT may offer a way out of this, as the released males will be infected with *Wolbachia* and thus a lower irradiation dose can be applied that will allow for more competitive males. This combined strategy could in principle be applied to any targeted species for which an adequate sexing system is not available. Integration of such a protocol combining low irradiation dose with CI has proved to be an efficient strategy in programs targeting the population suppression of *Aedes albopictus* (Zhang et al. 2015a, b, 2016).

## Requirements for SIT and/or IIT

Before the application of an SIT and/or IIT program against *D. suzukii,* it is, nevertheless, important to consider potential limiting factors that may render the program ineffective.

### Laboratory domestication and mass rearing

Apart from the factors affecting mass rearing, the process itself requires industrial-scale equipment and protocol that will allow for the mass production of high-quality sterile *D. suzukii* insects in a cost-effective manner. Another challenge is to develop an economic viable artificial larval and adult diet. The quality of the sterile insects should be continuously monitored to ensure that the desirable biological traits are maintained.

Rearing is a crucial step for SIT and IIT, and the initial fly material used in the rearing process as well as the genomic changes/adaptations is important factors regarding biological quality and consequently release of the manipulated fly specimens (Gilchrist et al. 2012). It is known that within some generations populations can adapt to the mass-rearing environment producing individuals which may significantly differ from their wild counterparts (Gilchrist et al. 2012; Gilligan and Frankham 2003). Several life history traits could be affected during the laboratory adaptation process including reduction in developmental time, lifespan, dispersal ability and stress resistance, as well as early fertility and increased fecundity (Gilchrist et al. 2012; Hoffmann et al. 2001; Raphael et al. 2014). Several studies have reported that genetic diversity loss may occur rapidly during the early generations of domestication (Gilchrist et al. 2012; Raphael et al. 2014; Zygouridis et al. 2014). Results from Gilchrist et al. (2012) in *Bactrocera tryoni* concur with other studies in *Bactrocera oleae* documenting the loss of genetic diversity in captive populations (Zygouridis et al. 2014). This genetic issue and the associated phenotypic effects may severely compromise the success of SIT and IIT programs as the fly quality in the *D. suzukii* mass-rearing facility may be severely jeopardized. Thus, it is required to develop a strategy that will allow maintaining genetic diversity, biological quality and competiveness.

Symbiotic bacteria have shown to affect several aspects of the biology, physiology, nutrition and ecology including reproduction and mating behavior of their insect hosts in diverse ways (Augustinos et al. 2015; Bourtzis and Miller 2003, 2006, 2009; Douglas 2011; Eleftherianos et al. 2013; Engel and Moran 2013; Koukou et al. 2006; Miller et al. 2010; Sharon et al. 2010; Weiss and Aksoy 2011; Zchori-Fein and Bourtzis 2011). *Drosophila* species is associated with taxonomically restricted microbial communities compared to mammals, with only four bacterial families being the dominant taxa (Broderick and Lemaitre 2012; Chandler et al. 2011; Corby-Harris et al. 2007; Erkosar et al. 2013; Wong et al. 2013). Several factors influence the microbiota composition including environmental conditions and habitats, life cycle stages, host age and more especially diet (Chandler et al. 2011; Erkosar et al. 2013; Staubach et al. 2013; Yun et al. 2014). Diet proved to be a driving factor in shaping the gut microbiome diversity (Chandler et al. 2011). A specific diet determines which microbes are able to colonize this environment. As a result, most of the bacteria characterized in laboratory-reared *Drosophila* populations are not the most abundant in wild populations, and vice versa (Chandler et al. 2011; Staubach et al. 2013). These observations may explain why the fitness of some laboratory-adapted populations is not comparable to that of natural populations.

The biological quality of the mass-produced insects is of major importance for SIT and IIT applications, and its improvement in a mass-rearing facility would result in enhancement of the efficacy of SIT and/or IIT applications. Mass rearing and/or irradiation may affect the gut bacterial community structure of insects, and this may also impact mating competitiveness of sterile males (Ben Ami et al. 2010; Hamden et al. 2013). Similarly, several studies have shown that *Wolbachia* may be associated with mating isolation phenomena (Koukou et al. 2006; Miller et al. 2010). Since insect-associated microbiota seems to play a major role in fly quality, it is important to identify the factors that could alter and/or modify the composition of the intestinal symbionts and consequently reduce the overall fitness of the sterile males. In addition, the use of probiotics (originating from endogenous gut-associated bacterial species) should be explored, as a means to improve rearing efficiency and mating competitiveness as shown for medfly (Augustinos et al. 2015; Gavriel et al. 2011).

### Irradiation protocol

Irradiation dose required for complete male sterility may affect biological quality and mating competitiveness of *D. suzukii*. The optimal conditions, developmental stage and dose for irradiation-induced male sterility should be determined to minimize potential negative effects, while the use of probiotics could ameliorate them as shown for medfly (Gavriel et al. 2011). Applying the SIT to *D. suzukii* involves irradiation during a narrow time window at the late pupal stage to induce atrophy of the reproductive organs, therefore inducing reproductive sterility without affecting reproductive behavior, and then release into the target area where the sterile males sexually compete with their wild counterparts. An irradiation protocol must be thoroughly developed and tested to ensure a high degree of confidence that the process will properly sterilize the insects. For the combined SIT/IIT approach, it is also important to determine the minimum optimal dose for complete sterilization of female *D. suzukii* that will not influence the male mating competitiveness. Therefore, it is important that this dose is significantly below that normally required for full male sterility (Lees et al. 2015; Zhang et al. 2016).

The SIT and IIT programs also need to ensure that, once in the field, the sterile males compete effectively with wild males and mate with wild females and successfully transfer their sperm. Effective methods for monitoring and providing timely feedback on the quality and competitiveness of sterile fruit flies are critical to the success of SIT programs. The quality of the sterile mass-reared insects and the mating competitiveness—as measured by their ability to induce sterility in field females—with the wild counterparts are critical factors that should be measured and assessed using appropriate procedures (Dyck et al. 2005). Quality control protocols at laboratory and semi-field conditions level are required for the evaluation of flight ability and effectiveness of the mass-reared, irradiated and released sterile *D. suzukii*. The Joint FAO/IAEA Insect Pest Control Laboratory has developed a quality control manual for fruit flies (FAO/IAEA/USDA 2014). The manual includes procedures for product quality control (QC) for mass-reared and sterilized flies as well as handling and packaging methods of pupae intended to be used in SIT programs. These procedures involve series of tests that measure pupae weight, emergence, sterility, longevity, flight ability, sexual competitiveness and survival under stress. The quality of the mass-produced sterile insects will determine the ratio required for the population suppression.

### Management of thermal tolerance

Low-temperature treatment is an integral part of the rearing or release protocols in IPM programs (Colinet and Boivin 2011; Enkerlin 2007). For both SIT and IIT, efficient deployment of insects is achieved when their release coincides with the appearance of the pests, and there is often a timing gap between production and release. The ability to cold-store insects without loss of performance and to mobilize them quickly upon demand is thus essential for a viable biological control using SIT and/or IIT. Mass-reared insects are often exposed to low temperature for immobilization and handling during rearing process. Long-distance shipping from rearing facilities to release sites is also performed under low temperature. Finally, temperature within the release site (e.g., greenhouse) may contrast with rearing conditions and may be stressful to released insects (being too high or too low). This latter issue can be mitigated by application of thermal conditioning protocols before release to prevent thermal stress-induced mortality. In consequence, a successful application of SIT and/or IIT requires large basic knowledge on thermal biology of the target insect in order to develop protocols to manipulate its thermal tolerance.

Most recent studies on *D. suzukii* cold tolerance were designed to understand overwintering strategy in newly infested cold regions, in order to better predict invasion potential or winter survival probability (Dalton et al. 2011; Rossi-Stacconi et al. 2016; Shearer et al. 2016; Stephens et al. 2015; Wallingford and Loeb 2016; Zerulla et al. 2015). The recent literature shows that *D. suzukii* is freeze intolerant and chill susceptible (Dalton et al. 2011; Jakobs et al. 2015; Kimura 2004; Plantamp et al. 2016; Ryan et al. 2016), but possesses a large thermal tolerance plasticity, which likely favors its overwintering success (Jakobs et al. 2015). This large plasticity could be exploited to modulate *D. suzukii* thermal tolerance via classical acclimation protocols, e.g., pre-exposure to sublethal conditions (Colinet and Hoffmann 2012). *Drosophila suzukii* is supposed to overwinter as adult dark winter morph (Kanzawa 1936; Shearer et al. 2016; Stephens et al. 2015; Toxopeus et al. 2016; Wallingford et al. 2016). This morph is characterized by an arrest of reproduction and an increased cold tolerance (Shearer et al. 2016; Stephens et al. 2015; Toxopeus et al. 2016; Wallingford and Loeb 2016), but it is not yet clear whether this morph entails a true reproductive diapause or not (Rossi-Stacconi et al. 2016; Toxopeus et al. 2016; Wallingford et al. 2016; Zhai et al. 2016). Understanding how to initiate and arrest its diapause may provide valuable tools for long-term storage. Knowledge of the overwintering biology of *D. suzukii* is also a crucial factor in predicting the size of the summer population in a given area (Rossi-Stacconi et al. 2016). This will allow for more efficient planning of the control methods implementation. For instance, the SIT and IIT approaches that are based on the release of mass-produced sterile males could take advantage of the shortage of wild males during late winter/early spring periods. Their implementation at those periods would reduce the competition from the wild males and increase the mating frequency for the sterile males (Rossi-Stacconi et al. 2016). Given the high level of inconsistency in the available data regarding *D. suzukii* mortality at different temperatures, detailed thermo-biological data are highly needed (Asplen et al. 2015). To fully appreciate the innate capacity of *D. suzukii* to cope with both cold and heat stress, a comprehensive approach based on tolerance landscape has been proposed by Enriquez and Colinet (2017). The authors found that temperatures below 5–7 °C were likely not compatible with cold storage, while temperatures above 32 °C would drastically reduce survival. Thermal survival patterns were also influenced by sex, stage, as well as relative humidity. This basic information is important to develop protocols to manipulate *D. suzukii* thermal tolerance. Undoubtedly, studying thermal biology of *D. suzukii* is essential to facilitate the application of SIT and/or IIT, but other aspects of SIT and/or IIT could be impacted (positively or negatively) by temperature. For instance, lowering metabolic rate with low temperature or promoting generic mechanisms of stress tolerance with pre-exposures may mitigate off-target irradiation damages and promote post-irradiation performance. Applying mild thermal stress (heat or cold) at some specific stage could trigger antioxidant defenses and lower oxidative damages resulting from irradiation. Such a hormesis approach (i.e., physiological conditioning) has been observed when applying short-term anoxic conditions before irradiation in the Caribbean fruit fly (López-Martínez and Hahn 2014). Besides, thermal conditions may directly affect *Wolbachia* load (Mouton et al. 2006, 2007), and consequently, it is important to analyze whether thermal treatments allow maintenance of high level of CI. On the other hand, data coming from the study of Neelakanta et al. (2010) have shown that gut symbionts can enhance the ability of insects to tolerate cold temperatures and overwinter. Acquiring knowledge on basal and induced thermal tolerance of irradiated and *Wolbachia*-infected *D. suzukii* will also help in defining accurate management practices based on SIT and/or IIT programs.

## Conclusions

*Drosophila suzukii* has spread across Europe and causes significant economic losses in commercial soft fruits. Its rapid invasive rate in the continent poses a challenge to the development of efficient monitoring and management tools. *Drosophila suzukii* is a highly prolific species with exceptional biological traits that contribute to its persistence in distinct geographic areas. This fact, combined with its extreme polyphagy behavior, suggests that an AW-IPM program is required for the effective control of the pest. Chemical control tactics are currently the most widespread method used to control *D. suzukii*. Insect resistance to chemicals, frequent applications of insecticides owing to *D. suzukii*’s short generation time and concerns about public health are considerable issues that have turned research toward non-chemical, environmentally sound and sustainable approaches. Investment in innovative biological control methods could lead to a reduction in *D. suzukii*’s populations not only in cultivated crops, but also in natural niches that are normally neglected by chemical approaches. Development of SIT for *D. suzukii*, as a component of an AW-IPM approach, is expected to significantly contribute to population management, especially in greenhouses and other confined areas, such as exclusion netting high tunnels. Driven by the encouraging results of SIT applications on other pests, research groups have focused their efforts on addressing the challenges related to the method. In the unlikely event that the irradiated sterile males present decreased or insufficient mating competitiveness, employment of IIT as a supplement to SIT is worth considering. This combined strategy could offer a way out of dead ends posed by each method individually. Management of thermal tolerance is a crucial factor that allows cold storage of insects without loss of their performance. Acquiring knowledge on thermal biology of *D. suzukii* will allow the deployment of thermal tolerance manipulation protocols. As soon as these protocols are mature, the rearing and release processes of SIT or the combined SIT/IIT approaches will profit from them and become more reliable. Whatever the method to be applied, there are several questions that need to be addressed beforehand and knowledge gaps to shed light on. To this aim, research should be carried out to deploy accurate and well-established management programs to decrease the impact of this invasive pest.

## Author contributions

KN and KB organized the review. KN wrote the review, and HC contributed in the “Management of thermal tolerance” part. All authors reviewed and provided constructive comments for this manuscript. All authors read and approved the final manuscript.
